# 

**Published:** 1852-09

**Authors:** 


### NOTICE TO CORRESPONDENTS.

Communications and Books for notice should be addressed to the Editors, care of Messrs. Lindsay & Blakiston.

Letters, &c., connected with the *business affairs* of the Journal should be addressed to the Publishers.

Papers for publication must be received *before* the 20th of the month, or they cannot appear in the forthcoming number.

The following Journals have been received in exchange:

Medical News. Philadelphia.
Buffalo Journal.
L'Union Medicale.
New York Medical Gazette.
The Boston Medical and Surgical Journal. (Weekly.)
Stethoscope and Virginia Medical Gazette.
Western Journal.
New Jersey Medical and Surgical Reporter.
Nordamericanischer Monatsbericht.
Transylvania Medical Journal.
Nashville Journal of Medicine and Surgery.
Southern Medical and Surgical Journal.
New Hampshire Journal of Medicine.
The London Lancet. (Weekly, London.)
The Medical Times and Gazette. (Weekly, London.)
Dublin Medical Press.
London Journal of Medicine.
Edinburgh Medical and Surgical Journal.
London Pharmaceutical Journal.
Edinburgh Monthly Journal.
Journal of Pharmacy.
North Western Medical Intelligencer.
American Journal of Insanity.

---

### BOOKS RECEIVED.

Report of the Ohio Lunatic Asylum for the year 1851.
Wood's Practice of Medicine. New Edition. From Lippincott & Grambo.
Cooper's Lectures on Surgery. From Blanchard & Lea.
Sketches of Brazil, by Dr. Dundas. From the Author.
Bartlett on Fevers. New Edition. From Blanchard & Lea.
Beck on Infant Therapeutics.
Green on Polypi of the Larynx.
Flint's Clinical Notes.

---

The foreign correspondents of the Examiner will please direct their Exchanges and other communications to the care of Mr. Thos. Delf, No. 12 Paternoster Row, London, or Mr. H. Bossange, 21 Bis, Quai Voltaire, Paris.

